# Tauopathy Analysis in P301S Mouse Model of Alzheimer Disease Immunized with DNA and MVA Poxvirus-Based Vaccines Expressing Human Full-Length 4R2N or 3RC Tau Proteins

**DOI:** 10.3390/vaccines8010127

**Published:** 2020-03-13

**Authors:** Juan García-Arriaza, María Q. Marín, Jesús Merchán-Rubira, Sara M. Mascaraque, Miguel Medina, Jesús Ávila, Félix Hernández, Mariano Esteban

**Affiliations:** 1 Department of Molecular and Cellular Biology, Centro Nacional de Biotecnología (CNB), Consejo Superior de Investigaciones Científicas (CSIC), 28049 Madrid, Spain; mquiros@cnb.csic.es (M.Q.M.); Sara.MorenoMascaraque@med.uni-duesseldorf.de (S.M.M.); 2 Department of Molecular Neuropathology, Centro de Biología Molecular “Severo Ochoa” (CBMSO), CSIC-UAM, 28049 Madrid, Spain; jmerchan@cbm.csic.es (J.M.-R.); jesus.avila@csic.es (J.Á.); 3 Network Center for Biomedical Research on Neurodegenerative Diseases (CIBERNED), 28031 Madrid, Spain; mmedina@ciberned.es

**Keywords:** Alzheimer, tau, poxvirus, MVA, P301S transgenic mice, pathology

## Abstract

Alzheimer’s disease (AD) is a neurodegenerative disorder characterized by a progressive memory loss and cognitive decline that has been associated with an accumulation in the brain of intracellular neurofibrillary tangles (NFTs) formed by hyperphosphorylated tau protein, and extracellular senile plaques formed by β-amyloid peptides. Currently, there is no cure for AD and after the failure of anti β-amyloid therapies, active and passive tau immunotherapeutic approaches have been developed in order to prevent, reduce or ideally reverse the disease. Vaccination is one of the most effective approaches to prevent diseases and poxviruses, particularly modified vaccinia virus Ankara (MVA), are one of the most promising viral vectors used as vaccines against several human diseases. Thus, we present here the generation and characterization of the first MVA vectors expressing human tau genes; the full-length 4R2N tau protein or a 3RC tau fragment containing 3 tubulin-binding motifs and the C-terminal region (termed MVA-Tau4R2N and MVA-Tau3RC, respectively). Both MVA-Tau recombinant viruses efficiently expressed the human tau 4R2N or 3RC proteins in cultured cells, being detected in the cytoplasm of infected cells and co-localized with tubulin. These MVA-Tau vaccines impacted the innate immune responses with a differential recruitment of innate immune cells to the peritoneal cavity of infected mice. However, no tau-specific T cell or humoral immune responses were detected in vaccinated mice. Immunization of transgenic P301S mice, a mouse model for tauopathies, with a DNA-Tau prime/MVA-Tau boost approach showed no significant differences in the hyperphosphorylation of tau, motor capacity and survival rate, when compared to non-vaccinated mice. These findings showed that a well-established and potent protocol of T and B cell activation based on DNA/MVA prime/boost regimens using DNA and MVA vectors expressing tau full-length 4R2N or 3RC proteins is not sufficient to trigger tau-specific T and B cell immune responses and to induce a protective effect against tauopathy in this P301S murine model. In the pursuit of AD vaccines, our results highlight the need for novel optimized tau immunogens and additional modes of presentation of tau protein to the immune system.

## 1. Introduction

Alzheimer’s disease (AD) is the most common neurodegenerative disorder and the biggest cause of dementia worldwide, representing one of the major causes of dependence, disability and mortality in elderly people [1]. It is characterized by an irreversible and progressive neural atrophy and memory loss [2] that is associated with two main pathological lesions in the brain: the presence of senile plaques and the accumulation of neurofibrillary tangles (NFTs), both leading to neural death [3,4].

Senile plaques are formed of abnormally folded β-amyloid peptides and play an important role in the development of the disease, mainly in familial Alzheimer’s disease (FAD) [5]. Therefore, β-amyloid peptides, presented at the extracellular space in monomeric or aggregated forms, have been suggested to be a suitable target for AD treatment [6]. Several active [7,8] or passive [9,10] anti-β-amyloid immunotherapy procedures have been shown to lower cerebral β-amyloid levels and improve cognition in animal models of AD, but clinical trials have yielded disappointing results [11,12], including a phase II clinical trial that was halted because of adverse effects like meningoencephalitis [13].

NFTs consist of helical filaments of hyperphosphorylated tau protein inside neurons. Although senile plaques of β-amyloid appear earlier than NFT pathology, they don’t correlate with the disease progression as they reach a plateau early in the symptomatic phase of the disease [14,15]. However, NFT pathology shows correlation with the stage of the disease, its clinical features and severity [16,17,18,19,20,21]. Therefore, it has been suggested that by the time tau pathology appears, β-amyloid therapy would be useless [22]. This possibility could explain the failure of β-amyloid therapy once the disease has developed, at later stages. Furthermore, the presence of extracellular toxic tau protein has been described [23]. Thus, based on these observations, several active and passive tau immunotherapeutic approaches have been tested in animal models and in phase I and II clinical trials with the aim of inducing anti-tau antibodies capable of clearing tau pathological species and eventually improve neuronal function [24]. Active immunotherapeutic approaches include the whole tau molecule, tau in aggregated or modified forms or synthetic tau peptides, such as ACI-35 [25] and AADvac1 [26]. Most of the passive immunotherapeutic approaches consist of monoclonal anti-tau antibodies, such as 8E12, RO7105705 and BIIB092 [27], and it has been recently described that anti-tau antibodies enter the brain [28,29] and can be internalized in neurons via Fcγ receptors [30]. Moreover, in addition to conventional vaccines, gene-based methods have been used to induce the expression of proteins to activate the cellular immune response upon delivery of DNA vectors [31].

Modified vaccinia virus Ankara (MVA) is a highly attenuated poxvirus vector extensively used in several preclinical and clinical trials as a vaccine candidate against numerous infectious diseases and cancer [32,33,34]. MVA is safe, well tolerated and expresses high levels of heterologous antigens, triggering potent immune responses against them [35]. Therefore, the use of MVA as a vector to express the human tau gene could be an encouraging approach to generate novel vaccines that might control AD progression.

Here, we describe the generation and characterization of two novel vaccine candidates against AD based on the MVA vector expressing either the human full-length 4R2N tau isoform protein or a 3RC tau protein containing 3 tubulin-binding motifs and the C-terminal region (termed MVA-Tau4R2N and MVA-Tau3RC, respectively). Both MVA-Tau vaccine candidates correctly expressed the corresponding tau proteins, were detected in the cytoplasm of infected cells co-localized with tubulin, and the recombinant viruses were highly stable in cell culture upon multiple passages. Both MVA-Tau vaccines impacted on the in vivo recruitment of innate immune cells in the peritoneal cavity of infected mice, although no tau-specific T and B cell immune responses were detected in mice immunized with the MVA-Tau vectors. Vaccination of transgenic P301S mice, a mouse model for tauopathies, with the combination of two vectors, a DNA-Tau as a priming component (DNA-au4R2N or DNA-Tau3RC) and MVA-au as a booster (MVA-Tau4R2N or MVA-Tau3RC) was not able to decrease significantly the levels of hyperphosphorylated tau, a clinical sign of AD, nor have a significant impact on the motor capacity and survival rate. Besides the failure to control the AD progression in this P301S mouse model, these results open the path to generate novel optimized tau immunogens able to induce a better tau antigen presentation to the immune system and to develop novel vaccination protocols that could control disease progression.

## 2. Materials and Methods

### 2.1. Ethics Statement

Female C57BL/6OlaHsd mice (6 to 8 weeks old) were purchased from Envigo Laboratories, stored in the animal facility of the Centro Nacional de Biotecnología (CNB) (Madrid, Spain), and the immunogenicity studies were approved by the Ethical Committee of Animal Experimentation (CEEA) of CNB-CSIC (Madrid, Spain) and by the Division of Animal Protection of the Comunidad de Madrid (PROEX 331/14). The efficacy animal studies in transgenic P301S mice were approved by the CEEA of Centro de Biología Molecular “Severo Ochoa” (CBMSO) (Madrid, Spain) and the Division of Animal Protection of the Comunidad de Madrid (PROEX 62/14). Animal procedures were performed according to international guidelines and to the Spanish law under the Royal Decree (RD 53/2013).

### 2.2. Cells

DF-1 cells (an immortalized chicken embryo fibroblast (CEF) cell line, ATCC catalog no. CRL-12203), primary CEF cells (obtained from specific-pathogen-free 11-day-old eggs; MSD, Salamanca, Spain), and HeLa cells (immortalized human epithelial cervix adenocarcinoma cells, ATCC^®^ CCL-2) were grown in complete Dulbecco’s modified Eagle’s medium (DMEM) (Gibco-Life Technologies, Carlsbad, CA, USA) supplemented with 10% heat-inactivated fetal calf serum (FCS) Gibco-Life Technologies, Carlsbad, CA, USA) for DF-1 and CEF cells or 10% newborn calf serum (NCS) (Sigma-Aldrich, St. Louis, MO, USA) for HeLa cells. Cell cultures were maintained at 37 °C in a humidified incubator containing 5% CO_2_. Cell lines were infected with viruses and after 1 h of adsorption, the virus inoculum was removed and DMEM-2% FCS or DMEM-2% NCS was added to the cell cultures.

### 2.3. Viruses

Parental virus used for the generation of recombinant MVA-Tau4R2N and MVA-Tau3RC vaccine candidates is a wild-type (WT) MVA (MVA-WT) modified by inserting the green fluorescent protein (GFP) gene into the vaccinia virus (VACV) thymidine kinase (TK) locus and by deleting the immunomodulatory VACV genes *C6L*, *K7R*, and *A46R* (termed MVA-Δ-GFP) [36,37,38]. To generate the MVA-Tau4R2N or the MVA-Tau3RC vaccine candidates the GFP insert of MVA-Δ-GFP was substituted by the full-length human tau gene (isoform Tau4R2N) or the human Tau3RC fragment containing 3 tubulin-binding motifs and the C-terminal region, respectively. We have also used as a control the MVA-WT. All MVAs were grown in primary CEF cells to obtain a master seed stock (P2 stock), purified through two cycles of sucrose-cushion sedimentation, and titrated, as previously described [35]. All MVAs were free of contamination with mycoplasma, bacteria or fungi.

### 2.4. Human Tau Antigens

In this study we used the full-length human tau gene (isoform Tau4R2N; GenBank accession number X14474.1), and a tau 3RC fragment containing three tubulin-binding motifs and the C-terminal region [39]. Both tau sequences were previously cloned in the mammalian plasmid expression vector pSG5 to generate pSG5-Tau4R2N and the pSG5-Tau3RC plasmids, respectively (also termed in this study DNA-Tau4R2N and DNA-Tau3RC, respectively) that correctly expressed the Tau4R2N and Tau3RC proteins [40,41].

### 2.5. Construction of Plasmid Transfer Vectors pCyA-Tau4R2N and pCyA-Tau3RC

The plasmid transfer vectors pCyA-Tau4R2N and pCyA-Tau3RC were constructed and used for the generation of recombinant viruses MVA-Tau4R2N and MVA-Tau3RC, respectively, allowing the insertion of the human tau genes in the TK locus of parental MVA-Δ-GFP by homologous recombination, following an infection/transfection procedure, as previously described [36,37,38,42]. The full-length human Tau4R2N or the Tau3RC genes present in the mammalian plasmid expression vectors pSG5-Tau4R2N and pSG5-Tau3RC were amplified by PCR (primers will be provided upon request) and inserted in the plasmid transfer vector pCyA-20 [42] to generate the pCyA-Tau4R2N and the pCyA-Tau3RC plasmid transfer vectors, respectively. Plasmid transfer vectors pCyA-Tau4R2N and pCyA-Tau3RC contains the VACV synthetic early/late (sE/L) promoter, a multiple-cloning site where the human Tau4R2N or Tau3RC genes are inserted between the VACV TK-L and TK-R flanking regions, the selectable marker gene for ampicillin, and a β-galactosidase (β-Gal) reporter gene sequence between two repetitions of the VACV TK-L flanking arms that will lead the deletion of the β-galactosidase gene from the final recombinant virus by homologous recombination after successive passages. The correct generation of pCyA-Tau4R2N and pCyA-Tau3RC was confirmed by DNA sequence analysis.

### 2.6. Generation of Recombinant Viruses MVA-Tau4R2N and MVA-Tau3RC

MVA-Tau4R2N and MVA-Tau3RC were generated using MVA-Δ-GFP as parental virus and pCyA-Tau4R2N or pCyA-Tau3RC as plasmid transfer vectors, respectively, using an infection/transfection protocol previously described [36,37,38,42]. The MVA-Tau4R2N and MVA-Tau3RC recombinant viruses obtained were then grown in CEF cells, purified and titrated by plaque immunostaining assay [35].

### 2.7. Characterization of MVA-Tau4R2N and MVA-Tau3RC

#### 2.7.1. PCR

The correct generation and purity of recombinant viruses MVA-Tau4R2N and MVA-Tau3RC was confirmed by PCR with primers TK-L and TK-R, annealing in the VACV TK locus and allowing the amplification of the full-length human Tau4R2N or the Tau3RC inserts, as previously described [36,37,38,42]. Moreover, the correct presence of deletions in VACV *C6L*, *K7R* and *A46R* genes in MVA-Tau4R2N and MVA-Tau3RC was confirmed by PCR, as previously described [36,37,38,43,44]. Furthermore, the correct insertion of human Tau4R2N or Tau3RC genes was also confirmed by DNA sequence analysis.

#### 2.7.2. Western Blot

To confirm the correct expression of the human Tau4R2N or Tau3RC antigens present in MVA-Tau4R2N or MVA-Tau3RC, DF-1 cells were mock-infected or infected with MVA-Tau4R2N, MVA-Tau3RC, and MVA-Δ-GFP at 5 plaque forming units (PFUs)/cell and at 3, 6 and 24 h postinfection (hpi), cell extracts or supernatants were collected, lysed in Laemmli buffer, fractionated in 10% sodium dodecyl sulfate–polyacrylamide gel electrophoresis (SDS-PAGE), transferred to a nitrocellulose membrane and analyzed by Western blotting with a mouse monoclonal antibody against the microtubule-binding region of the human tau protein (antibody 7.51; kindly provided by Dr. C. M. Wischik, Aberdeen, UK; diluted 1:100) to evaluate the expression of the human Tau4R2N or Tau3RC proteins. As loading controls, a rabbit anti-β-actin antibody (Cell Signaling, Danvers, MA, USA; diluted 1:1000), and a rabbit anti-VACV E3 antibody (CNB; diluted 1:1000) were used. The membranes were incubated with the antibodies at 4 °C overnight. Anti-mouse horseradish peroxidase (HRP)-conjugated antibody (Sigma-Aldrich, St. Louis, MO, USA; diluted 1:2000), or anti-rabbit HRP-conjugated antibody (Sigma-Aldrich, St. Louis, MO, USA; diluted 1:5000), were used as secondary antibodies. The immunocomplexes were detected using an HRP-luminol enhanced-chemiluminescence (ECL) system (ECL Plus) (GE Healthcare, Chicago, IL, USA).

#### 2.7.3. Virus Growth

To determine the virus growth profile of MVA-Tau4R2N and MVA-Tau3RC, in comparison to that of parental MVA-Δ-GFP, DF-1 cells were infected in duplicate at 0.01 PFUs/cell with MVA-Δ-GFP, MVA-Tau4R2N or MVA-Tau3RC, as previously described [36,37,38,42]. At different times (0, 24, 48, and 72 hpi) cells were harvested and virus titers in cell lysates were determined by a plaque immunostaining assay in DF-1 cells, as previously described [35].

### 2.8. Genetic Stability of MVA-Tau4R2N and MVA-Tau3RC

The genetic stability of recombinant viruses MVA-Tau4R2N and MVA-Tau3RC was analyzed as previously described [36,37,38,42] during 9 low multiplicity of infection (MOI) serial passages by checking by Western blotting the expression of human Tau4R2N and Tau3RC proteins, as described above in Section 2.7.2.

### 2.9. Analysis of the Expression of Human Tau4R2N and Tau3RC Proteins by Confocal Immunofluorescence Microscopy

Immunofluorescence studies were done in HeLa cells mock-infected or infected at a MOI of 0.5 PFUs/cell with MVA-Tau4R2N, MVA-Tau3RC or MVA-WT for 24 h, as previously described [36,37,38,45]. We used as a microtubule marker a rabbit polyclonal anti-tubulin antibody (BioNova, Madrid, Spain; diluted 1:200), and to detect the human tau proteins we used a mouse monoclonal antibody against the microtubule-binding region of the human tau protein (antibody 7.51; diluted 1:200). Anti-tau and anti-tubulin antibodies were then detected with mouse or rabbit secondary antibodies conjugated with the fluorochrome Alexa Fluor 488 (green) and Alexa Fluor 594 (red), respectively (Invitrogen, Carlsbad, CA, USA; diluted 1:500). The cell nuclei were stained with 4′,6′-diamidino-2-phenylindole (DAPI) (Sigma-Aldrich, St. Louis, MO, USA). Images of sections of the cells were acquired using a Leica TCS SP5 microscope and were recorded and processed.

### 2.10. Recruitment of Immune Cells in the Peritoneal Cavity of C57BL/6 Mice Inoculated with MVA-Tau4R2N and MVA-Tau3RC

Groups of female C57BL/6OlaHsd mice (6 to 8 weeks old; *n* = 5 mice per group) were injected by the intraperitoneal (i.p.) route with 1 x 10^7^ PFUs per mouse of MVA-Tau4R2N, MVA-Tau3RC, MVA-∆-GFP or PBS. At 24 and 48 h post inoculation, peritoneal exudate cells were collected in 6 mL of PBS-2% FCS and the presence of different immune cells was analyzed by flow cytometry, as previously described [37,46,47]. Absolute numbers of immune cell populations for each mouse were determined by flow cytometry after extrapolation to the number of cells counted after the peritoneal washes.

### 2.11. P301S Transgenic Mice Immunization Schedule

The efficacy of the recombinant viruses MVA-Tau4R2N and MVA-Tau3RC was evaluated in transgenic P301S mice, a mouse model for tauopathies, obtained from Jackson laboratory (B6;C3-Tg(Prnp-MAPT*P301S)PS19Vle/J), that carries a mutant (P301S) human microtubule-associated protein tau (MAPT) gene encoding T34-tau isoform (1N4R) driven by the mouse prion-protein promoter (Prnp) on a B6C3H/F1 genetic background [21,48]. This background of P301S mice was homogenyzed to C57BL/6 by backcrossing these mice with C57BL/6 wild-type females in our laboratory. For the study of the efficacy of MVA-Tau4R2N, groups of transgenic P301S mice (*n* = 4 mice/group; male and female of 13 weeks of age at the beginning of the study) were immunized with 100 µg of pSG5-Tau4R2N (termed DNA-Tau4R2N) or pSG5-Φ (termed DNA-Φ), by the intramuscular (i.m.) route. Four weeks after the first immunization (week 17), mice received a booster dose with 2 × 10^7^ PFU of MVA-Tau4R2N or MVA-WT by the i.p. route. Additionally, 4 C57BL/6 WT mice were immunized at the same time points (weeks 13 and 17) with PBS. At week 31 mice were sacrificed and their brains were collected to study the tau phosphorylation by immunohistochemistry or Western blot (see Section 2.12). For the study of the efficacy of MVA-Tau3RC, groups of transgenic P301S mice (*n* = 7 mice/group; male and female of 22 weeks of age at the beginning of the study) were immunized with 100 µg of pSG5-Tau3RC (termed DNA-Tau3RC) or DNA-Φ by the i.m. route. Four weeks after the first immunization (week 26), mice received a booster dose with 2 × 10^7^ PFU of MVA-Tau3RC or MVA-WT by the i.p. route. At weeks 34, 41, and 47 (months 8, 9.5 and 11, respectively) the motor capacity of the mice was evaluated by a rotarod test (see Section 2.13). At week 48, mice were sacrificed and their brains were collected to study the tau phosphorylation by western blot or immunohistochemistry (see Section 2.12).

### 2.12. Study of Tau Phosphorylation in Brain Samples by Western Blot or Immunohistochemistry

Tau phosphorylation in hippocampal brain samples was analyzed by Western blotting, as previously described [49]. Extracts were prepared by homogenizing hippocampal samples in ice-cold extraction buffer consisting of 50 mM Tris-HCl pH 7.4, 150 mM NaCl, 1% NP-40, 1 mM sodium orthovanadate, 1 mM EDTA, a protease inhibitor cocktail (cOmplete™, Roche), and 1 μM okadaic acid. Protein content was determined by the Bradford protein assay (Sigma-Aldrich, St. Louis, MO, USA) and 20 μg of total protein were electrophoresed on a 10% SDS-PAGE and then transferred to a nitrocellulose membrane. Prior to antibody binding, membranes were blocked with 5% nonfat dried milk. To evaluate the expression of total and phosphorylated human tau proteins, a mouse monoclonal antibody against total human tau protein (antibody TAU-5; Merck Millipore, Burlington, MA, USA; diluted 1/1000), and a mouse monoclonal antibody that recognizes tau protein phosphorylated at both serine 202 and threonine 205 (antibody AT8; Thermo Fisher Scientific, Waltham, MA USA; diluted 1/100) were used. As loading controls, a mouse anti-glyceraldehyde-3-phosphate dehydrogenase (GADPH) antibody (Abcam; diluted 1/5000), or a mouse anti β-actin antibody (Sigma-Aldrich, St. Louis, MO, USA; diluted 1/1000) were used. The membranes were incubated with the antibodies at 4 °C overnight. An anti-mouse HRP-conjugated antibody (Dako; diluted 1/5000) was used as secondary antibody, and the immunocomplexes were detected using an ECL system (Amersham Biosciences, Little Chalfont, UK) and analyzed in a ChemiDoc—Imaging System (Bio-Rad, Hercules, CA, USA). Band intensities were quantified using ImageJ software (NIH, Bethesda, MD, USA) and phospho-Tau (AT8)/Tau (TAU-5) intensity ratios were represented and analyzed using GraphPad Prism Software (San Diego, CA, USA).

Tau phosphorylation in dorsal dentate gyrus samples was analyzed by immunohistochemistry, as previously described [49]. Sections were immersed in 0.3% H_2_O_2_ in PBS for 30 min to quench endogenous peroxidase activity. Subsequently, sections were blocked for 1 h in PBS containing 0.5% Fetal Bovine Serum, 0.3% Triton X-100 and 1% BSA (Sigma-Aldrich, St. Louis, MO) and incubated overnight at 4 °C in PBS containing 0.3% Triton X-100 and 1% BSA with the corresponding primary antibody: anti-tau TAU-5 (Calbiochem, San Diego, CA, USA) or anti-phosphorylated tau AT8 (Thermo Fisher Scientific) antibodies. Finally, brain sections were incubated with anti-mouse secondary antibody and avidin-biotin complex using the Elite Vectastain kit (Vector Laboratories). Chromogen reactions were performed with diaminobenzidine (SIGMAFASTTM DAB, Sigma-Aldrich, St. Louis, MO, USA) for 10 min. Mouse sections were mounted on glass slides and coverslipped with Mowiol (Calbiochem). Images were captured using an Olympus BX41 microscope with an Olympus camera DP-70 (Olympus Denmark A/S).

### 2.13. Rotarod Test

Motor coordination and balance of immunized P301S mice were tested by rotarod test by using an accelerating rotarod apparatus (Ugo Basile, Comerio, Italy). After a pre-trained period of two days at a constant speed (4 rpm the first day over 1 min four times and 8 rpm over 1 min four times the second day), on the third day the rotarod accelerated from 4 to 40 rpm over 5 min and mice were tested three times. The latency to fall was measured during accelerating trials.

### 2.14. Data Analysis and Statistical Procedures

The statistical significance of differences between groups in the experiment of cell recruitment was determined by Student’s t test (unpaired, non-parametric, two-tailed). The statistical analyses of the tau phosphorylation measured by immunohistochemistry and Western blotting in immunized P301S mice were done using one-way ANOVA (unpaired, two-tailed) with Dunnett’s correction for multiple comparisons. Statistical analysis of rotarod test was performed using Student’s t test. For the survival analysis, we carried out a Kaplan–Meier comparison of the survival curves (Log-rank Mantel-Cox test). Significant differences are described as follows: * *p* ≤ 0.05; ** *p* ≤ 0.005; *** *p* ≤ 0.001.

## 3. Results

### 3.1. Generation and In Vitro Characterization of MVA-Tau4R2N and MVA-Tau3RC

**Figure 1:**
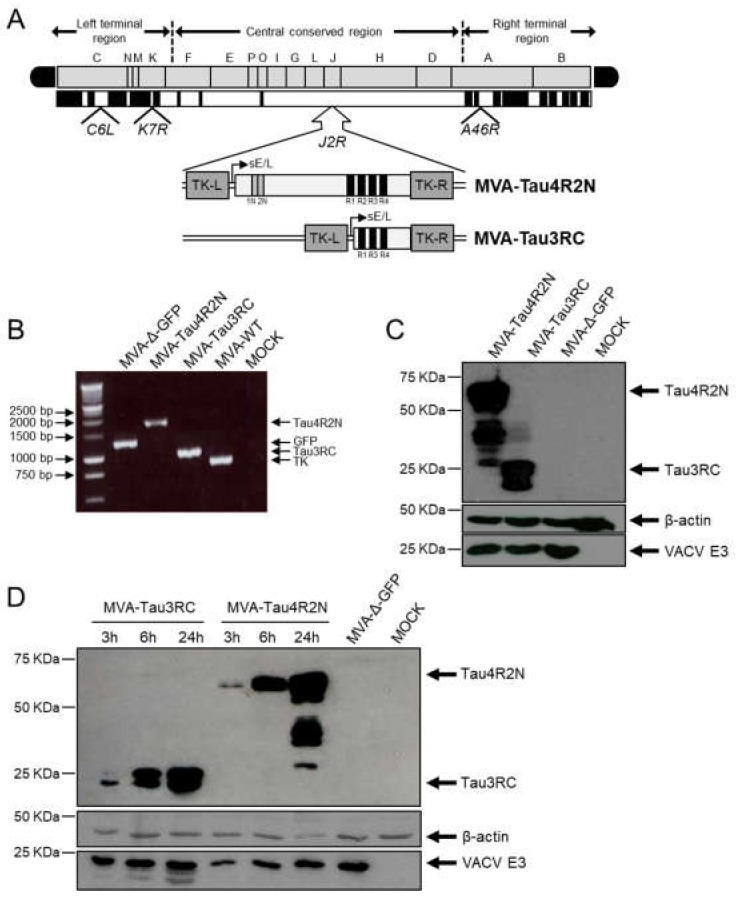
Generation and in vitro characterization of MVA-Tau4R2N and MVA-Tau3RC vaccine candidates. (**A**) Scheme of the MVA-Tau4R2N and MVA-Tau3RC genome map. The full-length human tau gene (isoform Tau4R2N, including 2 N-terminal inserts and 4 microtubule-binding repeats) and the Tau3RC fragment (including 3 microtubule-binding repeats) are driven by the VACV sE/L promoter and are inserted within the VACV TK viral locus (*J2R*). The deleted VACV *C6L*, *K7R*, and *A46R* genes are indicated. TK-L, TK left; TK-R, TK right. (**B**) PCR analysis of the VACV TK locus. Viral DNA was extracted from DF-1 cells mock infected or infected at 5 PFU/cell for 24 h with MVA-Tau4R2N, MVA-Tau3RC, MVA-Δ-GFP, or MVA-WT. Primers spanning the TK locus-flanking regions were used for PCR analysis of the human Tau4R2N and Tau3RC genes inserted within the VACV TK locus. DNA products are indicated by an arrow on the right. A molecular size marker (1-kb ladder) with the corresponding sizes (base pairs) is indicated on the left. (**C**) Expression of human Tau4R2N and Tau3RC proteins. DF-1 cells were mock infected or infected at 5 PFU/cell with MVA-Tau4R2N, MVA-Tau3RC, or MVA-Δ-GFP. At 24 hpi, cells were lysed, fractionated by 10% SDS-PAGE, and analyzed by Western blotting. Arrows on the right indicate the positions of the full-length human Tau4R2N or the Tau3RC proteins (detected with the anti-tau antibody 7.51), VACV E3 protein or β-actin. The sizes of standards (in kDa) are indicated on the left. (**D**) Expression kinetics of human Tau4R2N and Tau3RC proteins. DF-1 cells were mock infected or infected at 5 PFU/cell with MVA-Tau4R2N, MVA-Tau3RC or MVA-Δ-GFP. At 3, 6 and 24 hpi expression of full-length human Tau4R2N and Tau3RC proteins, VACV E3 protein or β-actin was determined in cell extracts by western blot, as above.

To generate novel vaccines against AD that could impact on tau pathology, we have developed two MVA-based vaccine candidates expressing either the full-length human Tau4R2N isoform or the Tau3RC mutant protein (termed MVA-Tau4R2N and MVA-Tau3RC, respectively). Human Tau4R2N and Tau3RC genes were inserted in the vector backbone of an optimized parental MVA (termed MVA-Δ-GFP) that contains deletions in the VACV immunomodulatory genes *C6L*, *K7R*, and *A46R* [36,37,38], and were placed into the VACV TK locus under the transcriptional control of the VACV sE/L promoter driving the constitutive expression of the human tau proteins (Figure 1A). This optimized MVA vector expressing antigens from various pathogens was successfully used as a vaccine candidate against chikungunya virus (CHIKV) [36], Ebolavirus [37] and Zika virus [38] triggering broad T and B cell immune responses in animals and providing high protective efficacy after virus challenge.

The correct insertion and purity of recombinant MVA-Tau4R2N and MVA-Tau3RC viruses were analyzed by PCR using primers annealing in the VACV TK-flanking regions that confirmed the presence of the full-length human Tau4R2N and the Tau3RC genes in MVA-Tau4R2N and MVA-Tau3RC, respectively (Figure 1B). Moreover, the correct sequence of both full-length human Tau4R2N and Tau3RC genes inserted in the VACV TK locus was also confirmed by DNA sequencing.

To show that MVA-Tau4R2N and MVA-Tau3RC constitutively express the full-length human Tau4R2N and Tau3RC proteins, respectively, we carried out a Western blot analysis using the specific antibody 7.51 that binds to the microtubule-binding region to analyze cell extracts from DF-1 cells mock infected or infected at a MOI of 5 PFU/cell with MVA-Tau4R2N, MVA-Tau3RC, or parental MVA-Δ-GFP for 24 h. The results demonstrated that MVA-Tau4R2N and MVA-Tau3RC properly expressed the full-length human Tau4R2N and Tau3RC proteins, respectively (Figure 1C). Moreover, a kinetic time course showed that both MVA-Tau vaccine candidates expressed the human Tau4R2N and Tau3RC proteins in cell extracts as early as 3 hpi, reaching higher levels at 24 hpi (Figure 1D), although both proteins were not detected in the supernatant of infected cells.

**Figure 2:**
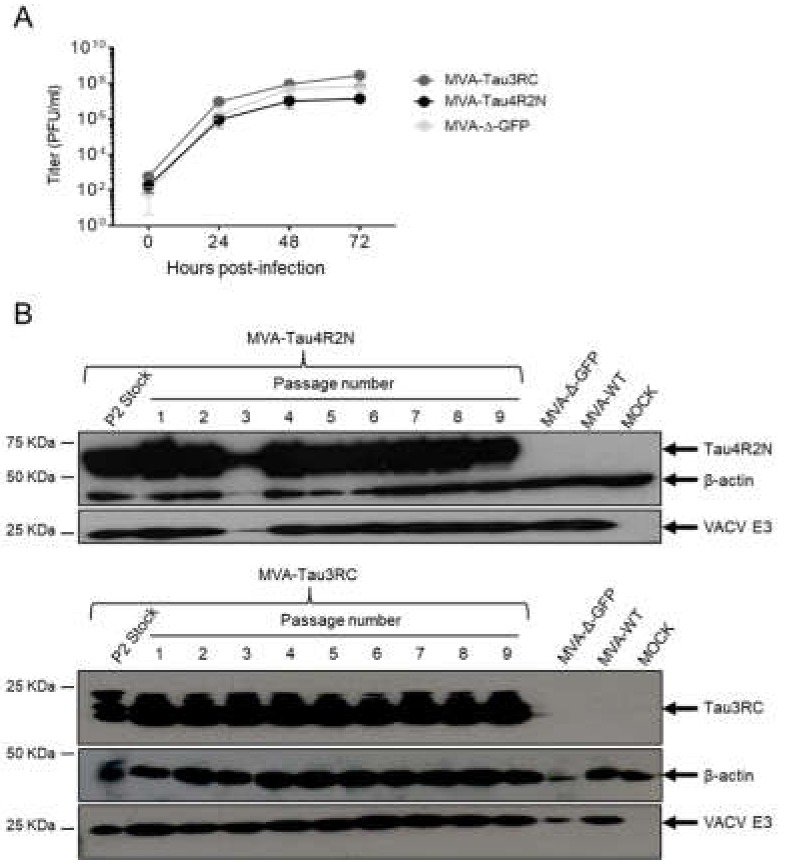
Viral growth kinetics and stability of MVA-Tau4R2N and MVA-Tau3RC. (**A**) Viral growth kinetics of MVA-Tau4R2N and MVA-Tau3RC. Monolayers of DF-1 cells were infected at 0.01 PFU/cell with MVA-Tau4R2N, MVA-Tau3RC, or MVA-Δ-GFP. At different times postinfection (0, 24, 48, and 72 hpi), virus titers in cell lysates were quantified by a plaque immunostaining assay. The mean and standard deviations from two independent experiments are shown. (**B**) Stability of MVA-Tau4R2N and MVA-Tau3RC. P2 stocks of MVA-Tau4R2N and MVA-Tau3RC were continuously grown at low MOI of 0.01 PFU/cell to passage 9 in DF-1 cells. The expression of Tau4R2N and Tau3RC proteins was determined after 3 days of infection by Western blotting using the anti-tau antibody 7.51. Rabbit anti-β-actin and anti-VACV E3 protein antibodies were used as a cellular and VACV loading controls, respectively. Arrows on the right indicate the position of the Tau4R2N and Tau3RC proteins, β-actin and the VACV E3 protein. The sizes of standards (in kDa) are indicated on the left.

Next, to determine whether expression of the full-length human Tau4R2N or Tau3RC proteins affects MVA replication in cell culture, we evaluated the growth kinetics of MVA-Tau4R2N and MVA-Tau3RC in DF-1 cells, in comparison to parental MVA-Δ-GFP. The results showed that the kinetics of viral growth were similar between all the viruses (Figure 2A), indicating that the constitutive expression of the full-length human Tau4R2N or Tau3RC proteins does not weaken MVA vector replication under permissive conditions.

Then, to ensure that MVA-Tau4R2N and MVA-Tau3RC are stable and can be maintained in cultured cells without the loss of the full-length human Tau4R2N or Tau3RC genes, both MVA-Tau vaccine candidates were additionally grown in DF-1 cells infected at a MOI of 0.01 PFU/cell during 9 consecutive passages, and expression of the human Tau4R2N or Tau3RC proteins during the different passages was determined by Western blotting. The results showed that both MVA-Tau vaccine candidates efficiently expressed the human tau proteins after successive passages, demonstrating that recombinant MVA-Tau4R2N and MVA-Tau3RC are genetically stable (Figure 2B).

### 3.2. The Human Tau4R2N and Tau3RC Proteins Expressed by MVA-Tau Vaccine Candidates are Located in the Cytoplasm and Co-Localize with Tubulin

**Figure 3:**
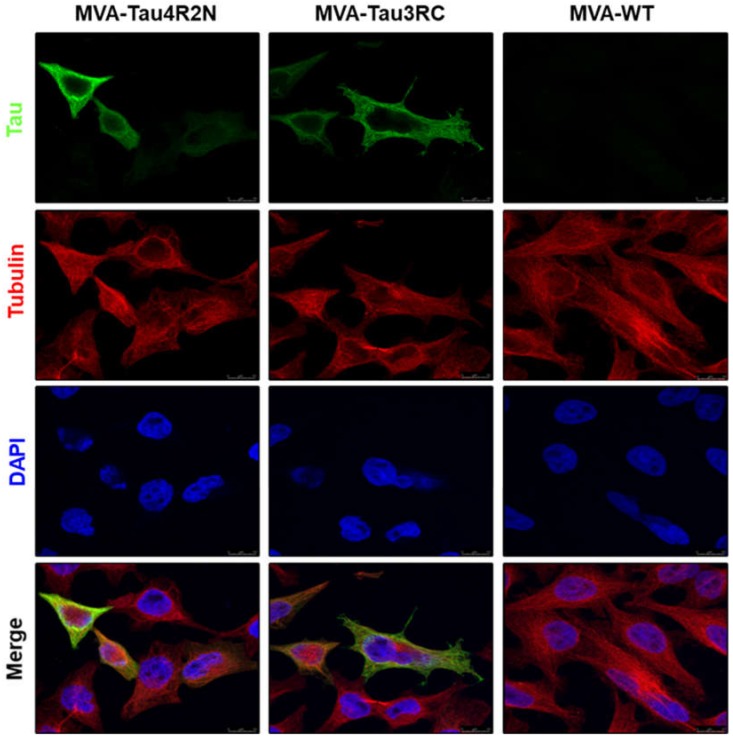
Immunofluorescence analysis of human tau proteins expressed by MVA-Tau4R2N and MVA-Tau3RC. HeLa cells were infected at 0.5 PFU/cell with MVA-Tau4R2N, MVA-Tau3RC, or MVA-WT for 24 h. Then, cells were fixed and permeabilized, followed by labeling with a mouse monoclonal anti-tau antibody (7.51), or a rabbit anti-tubulin antibody. Anti-tau was detected with a mouse secondary antibody conjugated with the fluorochrome Alexa Fluor 488 (green), and anti-tubulin was detected with a rabbit secondary antibody conjugated with Alexa Fluor 594 (red). Cell nuclei were stained using DAPI (blue). Scale bar: 10 μm.

The expression and intracellular localization of the human Tau4R2N and Tau3RC proteins expressed by MVA-Tau4R2N and MVA-Tau3RC was studied by confocal immunofluorescence microscopy in HeLa cells infected at a MOI of 0.5 PFU/cell with MVA-Tau4R2N, MVA-Tau3RC or MVA-WT. At 24 hpi cells were permeabilized and stained with antibodies against human tau protein and tubulin (Figure 3). The results showed that the human Tau4R2N and Tau3RC proteins (in green) were abundantly expressed in the cytoplasm of MVA-Tau4R2N- or MVA-Tau3RC-infected cells and co-localized with tubulin (in red).

### 3.3. MVA-Tau Vaccine Candidates Impact on the Recruitment of Immune Cells to the Peritoneal Cavity of Infected Mice

**Figure 4:**
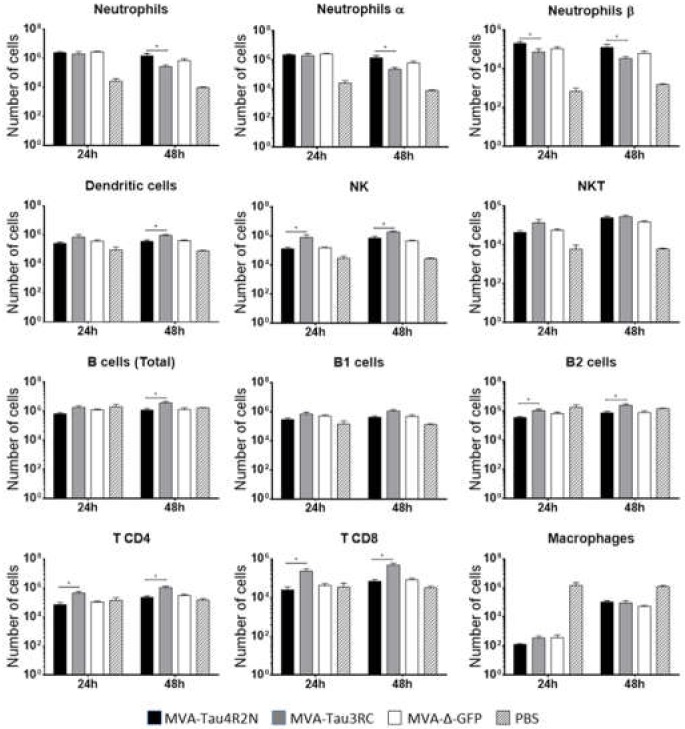
Recruitment of innate immune cells in the peritoneal cavity of mice infected with MVA-Tau4R2N and MVA-Tau3RC. Absolute numbers of innate immune cell populations obtained from the peritoneal cavity of C57BL/6 mice infected by the i.p. route with MVA-Tau4R2N, MVA-Tau3RC, MVA-Δ-GFP, or PBS. Peritoneal exudate cells were collected at 24 and 48 h post inoculation from each individual mouse (*n* = 5 per group), stained for different surface markers and absolute numbers were analyzed by flow cytometry. Graphs show the logarithmic mean ± SD, and statistical significance difference between MVA-Tau4R2N and MVA-Tau3RC groups are included (* *p* ≤ 0.05).

Next, to analyze whether the MVA-Tau vaccine candidates impact on the innate immune responses in vivo we analyzed the profile of immune cells that migrate to the peritoneal cavity after infection of mice with MVA-Tau4R2N and MVA-Tau3RC. Thus, we injected i.p. C57BL/6 mice with MVA-Tau4R2N, MVA-Tau3RC, MVA-Δ-GFP and PBS (1 × 10^7^ PFUs/mouse; five mice per group; see Materials and Methods), and determined by flow cytometry the absolute numbers of several immune cell populations present in the peritoneal cavity at 24 and 48 h post inoculation (Figure 4). The recruitment of immune cells was different between both MVA-Tau vaccine candidates and also compared to parental MVA-Δ-GFP. MVA-Tau3RC induced a significantly higher migration of natural killer (NK) cells, B2 cells, CD4 and CD8 T cells, than MVA-Tau4R2N and parental MVA-Δ-GFP at 24 h and 48 h, with also a significant enhancement in the recruitment of dendritic cells, and total B cells at 48 h. On the other hand, MVA-Tau4R2N induced a higher migration of neutrophils (total, α and β) than MVA-Tau3RC and parental MVA-Δ-GFP at 48 h, with an increased number of neutrophils β being also observed at 24 h. In summary, both MVA-Tau vaccine candidates impact differently on immune cell recruitment in the peritoneal cavity of infected mice, inducing a migration of innate immune cells.

### 3.4. MVA-Tau4R2N does not Reduce Significantly Hyperphosphorylated Tau in the Brains of Vaccinated Transgenic P301S Mice

**Figure 5:**
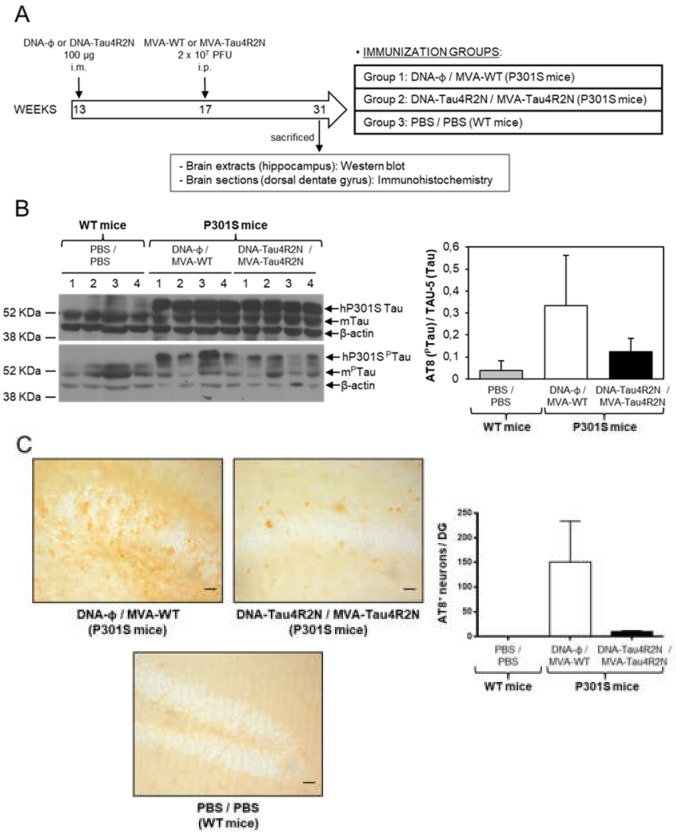
Studies on efficacy of MVA-Tau4R2N in transgenic P301S mice. (**A**) Vaccination scheme. Groups of transgenic P301S mice (*n* = 4 mice/group, 13 weeks of age) were immunized with 100 µg of DNA-Tau4R2N (pSG5-Tau4R2N) or DNA-Φ (pSG5) by the i.m. route. Four weeks later (week 17), animals were immunized with 2 × 10^7^ PFUs of MVA-Tau4R2N or MVA-WT by the i.p. route (see Materials and Methods). At week 31, animals were sacrificed and levels of hyperphosphorylated tau were determined by Western blot (**B**) and immunohistochemistry (**C**). (**B**) Detection of phosphorylated tau protein by Western blot in hippocampal samples of vaccinated P301S mice. Levels of human transgenic tau (hP301S) and endogenous murine tau (mTau) proteins were analyzed in hippocampal samples from C57BL/6 WT mice vaccinated with PBS/PBS and from transgenic P301S mice vaccinated with DNA-Φ/MVA-WT or DNA-Tau4R2N/MVA-Tau4R2N. TAU-5 (upper panel) and AT8 (lower panel) antibodies, were used to detect unphosphorylated and phosphorylated tau protein, respectively. β-actin has been used as a loading control. A quantification of all the bands of unphosphorylated and phosphorylated tau proteins detected by Western blot was made using ImageJ software, and the percentage of phosphorylated tau (^P^Tau) vs. unphosphorylated tau is shown in the graph, with error bars indicating the SEM. (**C**) Detection of phosphorylated tau protein by immunohistochemistry in dorsal dentate gyrus samples of vaccinated P301S mice. Immunohistochemistry was performed using the AT8 antibody to detect phosphorylated tau. Representative pictures from C57BL/6 WT mice vaccinated with PBS/PBS and transgenic P301S mice vaccinated with DNA-Φ/MVA-WT or DNA-Tau4R2N/MVA-Tau4R2N are shown. Scale bar: 20 μm. In the graph a quantification of the mean number of AT8^+^ cells per dorsal dentate gyrus (granule-cell layer) in 50-μm-thick sections is shown; error bars indicate the SEM.

Next, as a first proof-of-concept we studied the efficacy of MVA-Tau4R2N as a vaccine candidate against AD in transgenic P301S mice, a mouse model normally used to study tauopathies [21]. To perform the immunization studies we used a widely employed and potent vaccination procedure based on priming with a DNA vector and boosting with a poxvirus MVA vector that is able to trigger high levels of antigen-specific T and B cell immune responses [50]. Thus, P301S mice (*n* = 4 mice/group; 13 weeks old) were immunized by the i.m. route with DNA-Tau4R2N (or empty DNA-Φ as a control) and, 4 weeks later, boosted by the i.p. route with MVA-Tau4R2N or MVA-WT, as described in Materials and Methods and in Figure 5A. Furthermore, C57BL/6 WT mice (*n* = 4 mice; 13 weeks old) inoculated with 2 doses of PBS at weeks 13 and 17 were used as control animals. At week 31, animals were sacrificed and the levels of hyperphosphorylated tau in the brain were determined by Western blotting analysis in hippocampal samples (Figure 5B) and by immunohistochemistry in dorsal dentate gyrus samples (Figure 5C). The western blot results, using the phosphoTau-specific AT8 antibody, showed that although the DNA-Tau4R2N/MVA-Tau4R2N immunization decreased the levels of hyperphosphorylated tau, in comparison to animals immunized with DNA-Φ/MVA-WT (Figure 5B), the differences observed were not statistically significant. Additionally, the analysis by immunohistochemistry of the number of cells containing hyperphosphorylated tau in dorsal dentate gyrus samples, showed that DNA-Tau4R2N/MVA-Tau4R2N immunization decreased the number of cells containing hyperphosphorylated tau, in comparison to animals immunized with DNA-Φ/MVA-WT (Figure 5C), but again the differences observed were not statistically significant. In summary, immunization with DNA-Tau4R2N/MVA-Tau4R2N does not reduce significantly hyperphosphorylated tau protein in vaccinated P301S mice.

### 3.5. MVA-Tau3RC does not Control Motor Failure and Mortality of Vaccinated Transgenic P301S Mice, Neither Decrease the Levels of Hyperphosphorylated Tau

**Figure 6:**
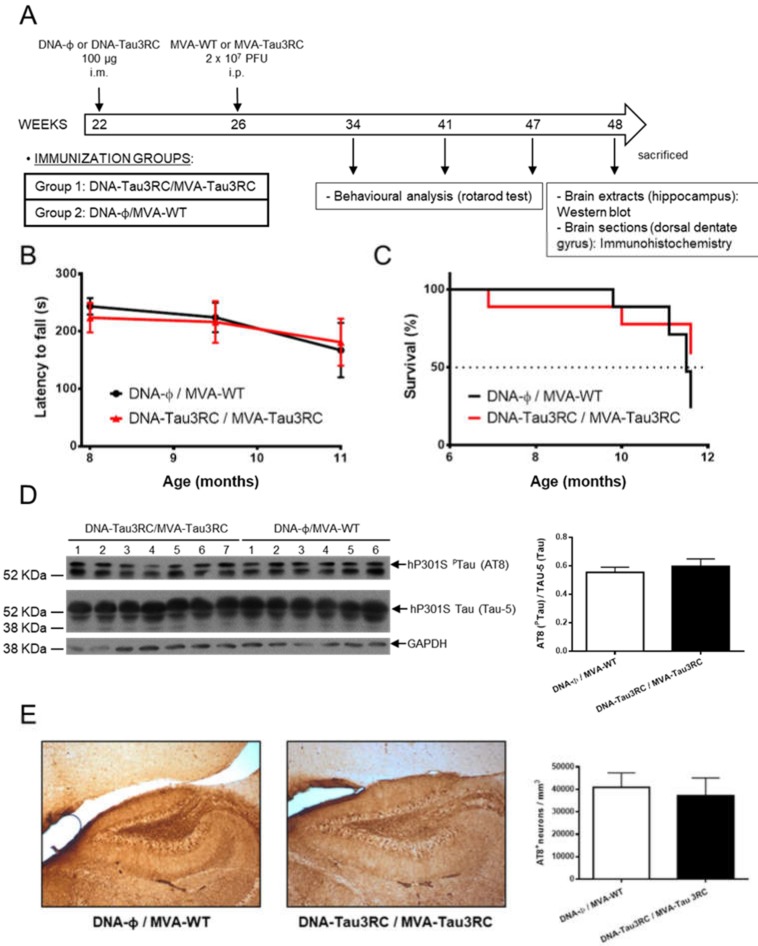
Studies on efficacy of MVA-Tau3RC in transgenic P301S mice. (**A**) Vaccination scheme. Groups of transgenic P301S mice (*n* = 6–7 mice/group, 22 weeks of age) were immunized with 100 µg of DNA-Tau3RC (pSG5-Tau3RC) or DNA-Φ (pSG5) by the i.m. route. Four weeks later (week 26), animals were immunized with 2 × 10^7^ PFUs of MVA-Tau3RC or MVA-WT by the i.p. route (see Materials and Methods). At weeks 34, 41, and 47 (months 8, 9.5 and 11, respectively) rotarod test were performed and animals were sacrificed at week 48. (**B**) Rotarod performance evolution. The latency to fall (in seconds) in P301S animals immunized with DNA-Tau3RC/MVA-Tau3C or DNA-Φ/MVA-WT was evaluated by a rotarod test at months 8, 9.5 and 11. The mean and SD are represented. (**C**) Survival rate. The percentage of P301S mice immunized with DNA-Tau3RC/MVA-Tau3C or DNA-Φ/MVA-WT surviving during the length of the experiment is represented, after the booster inoculation at week 26 (month 6). (**D**) Detection of phosphorylated tau protein by Western blot in hippocampal samples of vaccinated P301S mice. Levels of phosphorylated human tau (hP301S ^P^Tau) protein and total human tau (hP301S Tau) were analyzed in hippocampal samples from transgenic P301S mice vaccinated with DNA-Φ/MVA-WT or DNA-Tau3RC/MVA-Tau3RC. AT8 (upper panel) and TAU-5 (lower panel) antibodies were used to detect phosphorylated and unphosphorylated tau protein, respectively. GAPDH has been used as a loading control. A quantification of all the bands of unphosphorylated and phosphorylated tau proteins detected by Western blot was made using ImageJ software, and the percentage of phosphorylated tau (^P^Tau) vs. unphosphorylated tau (AT8/TAU-5) is shown in the graph, with error bars indicating the SEM. (**E**) Detection of phosphorylated tau by immunohistochemistry in dorsal dentate gyrus samples of vaccinated P301S mice. Immunohistochemistry was performed using the AT8 antibody to detect phosphorylated tau. Representative pictures from transgenic P301S mice vaccinated with DNA-φ/MVA-WT or DNA-Tau3RC/MVA-Tau3RC are shown. Scale bar: 20 μm. In the graph a quantification of the mean number of AT8 + cells per mm^3^ of dorsal dentate gyrus is shown; error bars indicate the SEM.

Next, we further analyze whether MVA-Tau3RC could control the tau pathology in vaccinated P301S mice by avoiding or diminishing the motor failure, increase survival rate and reduce the hyperphosphorylated tau. Thus, we vaccinated P301S mice (*n* = 7 mice/group; 22 weeks old) with DNA-Tau3RC (or empty DNA-Φ, as a control) and 4 weeks later (week 26) infected them with MVA-Tau3RC or MVA-WT, as described in Materials and Methods and Figure 6A. At weeks 34, 41, and 47 (months 8, 9.5 and 11, respectively), rotarod tests were performed to analyze the motor capacity of the mice (Figure 6B), and at week 48, mice were sacrificed. The survival rate was analyzed during the whole study (Figure 6C) and at week 48 the levels of hyperphosphorylated tau were determined by Western blot in hippocampal samples (Figure 6D) and by immunohistochemistry in dorsal dentate gyrus samples (Figure 6E).

The analysis of the motor capacity using rotarod tests showed that in all animals the latency to fall decreased with time, with no significant differences between mice immunized with DNA-Tau3RC/MVA-Tau3RC compared to DNA-Φ/MVA-WT control mice (Figure 6B). Furthermore, the analysis of the survival rate showed that at the end of the experiment (week 48) vaccinated mice had a survival rate (60%) higher than control mice (30%) (Figure 6C), but the differences observed were not significant.

The analysis by Western blotting of the hyperphosphorylated tau in hippocampal samples showed that both immunization groups induced similar levels of hyperphosphorylated tau (Figure 6D). Furthermore, the analysis by immunohistochemistry of the number of cells containing hyperphosphorylated tau in dorsal dentate gyrus samples also showed that DNA-Tau3RC/MVA-Tau3RC immunization group induced similar amount of cells with hyperphosphorylated tau than DNA-Φ/MVA-WT (Figure 6E). In summary, combined DNA-Tau3RC/MVA-Tau3RC immunization does not induce significant benefits in the motor behavior and survival rate nor in lowering hyperphosphorylation of tau protein in vaccinated P301S mice.

## 4. Discussion

A hundred years after Dr. Alzheimer documented for the first time the presence of tangles in a patient’s brain, we still lack a preventive or therapeutic treatment against AD that can successfully reduce the risk or delay the clinical phase of the illness. There have not been newly approved drugs against AD for over 15 years and immunotherapy represents a feasible approach against such a complex disease. Nowadays, there is a major effort in the pursuit of a vaccine against AD and targeting different AD-related antigens have been the main focus of research. As such, the two main targets are β-amyloid and tau. The therapeutic strategy that drives the development of vaccines against tau and β-amyloid protein is that the antibody enters the brain, binds to the protein and causes its subsequent elimination. However, one of the biggest challenges is that antibodies must cross the blood-brain barrier and reach their target. In this sense, an increase in the efficacy of the antibody can be achieved by increasing the natural transport systems of the blood-brain barrier [51]. Another factor to consider is the cellular location of the antigen, in our case the tau protein. The tau protein is an intracellular protein and the anti-tau antibodies enter in the brain [28,29] and can be internalized in neurons [30]. However, the most likely hypothesis is that its mechanism of action consists in eliminating extracellular tau and, therefore, blocking transcellular spreading [51]. However, preclinical and clinical studies against these two targets, mainly against β-amyloid, have not produced encouraging results [12]. These could be, in principle, due to the nature of the antigens or vaccine vectors used, the lack of achieving proper immune responses or the stimulation of the immune system in an undesirable manner, triggering proinflammatory responses with side effects. As yet, we still do not know what the main requirements for an effective immune response against AD are. Since many negative results were obtained for β-amyloid vaccination, we focused our study on tau protein, as it has been described that abnormal intracellular accumulation of hyperphosphorylated tau proteins forming NFTs is a pathological hallmark of AD and other related neurodegenerative disorders collectively termed tauopathies [52]. Therapies looking to decrease the amount of hyperphosphorylated tau gave negative results; thus, we are looking for alternative therapies [4,53]. Although there is no vaccine that can control AD progression, several tau immunotherapeutic approaches using active or passive immunizations have been developed [6,54,55] and are being tested in clinical trials.

Thus, based on the positive findings that have been obtained with poxvirus-based vectors, such as the eradication of smallpox and the use of recombinant poxvirus vectors in several preclinical and clinical trials as candidate vaccines against a wide spectrum of pathologies [32,33,34], we reasoned that using a potent immunization protocol for T and B cell activation based on combined DNA as a priming component followed by poxvirus MVA vectors as booster [50], we could develop a useful vaccination strategy, which in turn could provide important insights on how best to direct a more effective vaccination against AD. Hence, in this study we have generated two novel MVA-based vaccine candidates against AD that express either the full-length Tau4R2N protein or the Tau3RC mutant protein, termed MVA-Tau4R2N and MVA-Tau3RC, respectively. These MVA-based vaccine candidates were combined in a DNA prime/MVA boost approach with DNA-based vectors (DNA-Tau4R2N or DNA-Tau3RC) to define if any tau-specific immune response could be obtained and to evaluate whether a control in the disease progression could be observed in a mouse model of tauopathies, as a step toward developing more effective vaccine candidates.

MVA-Tau4R2N and MVA-Tau3RC vectors expressed high levels of the full-length Tau4R2N protein or the Tau3RC mutant protein in the cytoplasm of infected cells and, as expected, co-localized with tubulin, due to the presence of 4 or 3 microtubule-binding domains in the Tau4R2N or Tau3RC proteins, respectively. When both MVA-Tau vectors were injected in immunocompetent C57BL/6 mice by the i.p. route, they triggered a more efficient recruitment of dendritic cells, neutrophils, NK and NKT cells than the parental MVA vector, indicating that MVA-Tau4R2N and MVA-Tau3RC are able to activate the innate immune responses in vivo. It has been described that the innate immune response has a role in AD, and stimulation of the innate immune system via Toll-like receptor 9 (TLR9) agonists, such as type B CpG oligodeoxynucleotides (ODNs) is an effective and safe method to reduce tau-related pathology in AD mouse model [56,57].

The analysis of the tau-specific immunogenicity in immunocompetent BALB/c and C57BL/6 mice vaccinated with a DNA prime/MVA boost immunization protocol (either DNA-Tau4R2N/MVA-Tau4R2N or DNA-Tau3RC/MVA-Tau3RC) showed no tau-specific CD4^+^ or CD8^+^ T cells in the spleen of immunized mice at the peak of the response (10 days after the boost and following stimulation with tau peptides), although we could detect VACV-specific CD8^+^ T cells, reinforcing that the lack of an immune response is tau-specific. Moreover, binding antibodies against human tau protein were not detected in sera obtained from those immunized animals. The absence of tau-specific T-cellular and humoral immunogenicity could be due to a stabilization of the microtubule network induced by the larger human brain tau isoform (Tau4R2N) expressed by MVA-Tau4R2N or by the tau 3RC mutant protein expressed by MVA-Tau3RC, which would lead to an impairment of clonal expansion upon activation, finally resulting in a poor activation of the immune system of the mouse model, and a lack of function of the immune cells. Moreover, the presence of a microtubule-bound human tau protein may also result in the absence of a tau protein bound to the external membrane or the lack of a secreted tau protein, hence failing to be presented to T and B cells.

Furthermore, the efficacy study in the transgenic P301S mice showed that neither DNA-Tau4R2N/MVA-Tau4R2N nor DNA-Tau3RC/MVA-Tau3RC induced a significant reduction in the levels of hyperphosphorylated tau in the hippocampus and the dorsal dentate gyrus of vaccinated mice. Moreover, no control in the motor failure and mortality of vaccinated transgenic P301S mice was observed. To explain the failure to control the progression of the AD in immunized mice by both DNA-Tau and MVA-Tau vaccine candidates, we suggest that the expression of the human tau proteins (either Tau4R2N or Tau3RC) in immune cells may result in a loss of proliferation due to the microtubule stabilization promoted by these proteins. Thus, the presence of Tau4R2N or Tau3RC may prevent the microtubule depolymerization required for mitosis during cell proliferation. Therefore, future experiments will be performed to evaluate the expression by MVA of other tau isoforms or optimized tau fragments with a diminished microtubule stabilization, such as the C-terminal region of tau that has also been described as the most immunogenic region [55,58], or other regions as the N-terminal domain. Moreover, some tau fragments have been used with success in active immunization against AD [4,55], as well as a phosphorylated tau peptide bound to VLPs [29].

## 5. Conclusions

In conclusion, we described here for the first time the generation of novel vaccine candidates against AD based on MVA vectors expressing either the full-length human Tau 4R2N isoform (MVA-Tau4R2N) or the human tau 3RC mutant protein (MVA-Tau3RC). Well-established and potent prime/boost immunization protocols using DNA-Tau and MVA-Tau vaccine candidates did not induce tau-specific T-cell and humoral immune responses or a significant protection against AD-like disease in transgenic P301S mice. These results open the path to generate novel MVA-based vectors expressing optimized tau antigens that could elicit a better tau antigen presentation to the immune cells and to develop novel vaccination protocols that could control AD progression.
